# Parental preference for webcams in neonatal intensive care units: an indicator of lacking trust?

**DOI:** 10.1186/s12887-022-03456-2

**Published:** 2022-07-11

**Authors:** Laura Mause, Alinda Reimer, Jan Hoffmann, Till Dresbach, Dirk Horenkamp-Sonntag, Melanie Klein, Nadine Scholten, on behalf of Neo-CamCare, Nadine Scholten, Andreas Müller, Till Dresbach, Martin Hellmich, Christina Samel, Christiane Woopen, Christiane Jannes, Ludwig Kuntz, Indra Spiecker gen. Döhmann, Sebastian Bretthauer, Dirk Horenkamp-Sonntag, Stefanie Wobbe-Ribinski

**Affiliations:** 1 Institute of Medical Sociology, Health Services Research, and Rehabilitation Science, Faculty of Human Sciences and Faculty of Medicine, University of Cologne and University Hospital Cologne, Eupener Str. 129, 50933 Cologne, Germany; 2 University Hospital Bonn, Department of Neonatology and Pediatric Intensive Care, Children’s Hospital, University of Bonn, Bonn, Germany; 3 Techniker Krankenkasse, Healthcare Management, Hamburg, Germany; 4 DAK Gesundheit, Hamburg, Germany

**Keywords:** Neonatal intensive care unit, Parents, Parental needs, Trust, Webcam

## Abstract

**Background:**

Some neonatal intensive care units offer parents webcam systems for times when they cannot be in the ward. Leaving an infant in the ward can be challenging for parents, and trust in the neonatal healthcare professionals mitigates parents’ worries of not knowing how their infant is doing while they are away. If parents lack trust in the neonatal healthcare professionals, they may attempt to compensate by using webcams. In this work, we examine whether an association exists between the parental preference to use a webcam and low trust in physicians and nursing staff.

**Methods:**

In a nationwide, retrospective cross-sectional study, parents of infants with a birth weight below 1500 g were surveyed six to 18 months after their infant’s birth. Parents who were not offered a webcam system in the ward were asked whether they would have opted for it. Trust was measured by the Trust in Physician and Trust in Nursing Staff scales.

**Results:**

Of the parents who were not offered a webcam, 69% would have chosen to use a webcam if they had been granted the opportunity. The decision for or against a webcam was not significantly associated with either trust in physicians (OR = 0.654, 95% CI = 0.456, 0.937, *p* = .124) or trust in nursing staff (OR = 1.064, 95% CI = 0.783, 1.446, *p* = .932).

**Conclusions:**

While the majority of parents surveyed would opt for webcam usage, this preference should not be interpreted as an indicator of lacking trust in neonatal healthcare professionals.

**Supplementary Information:**

The online version contains supplementary material available at 10.1186/s12887-022-03456-2.

## Background

Premature infants with a very or extremely low birth weight are typically hospitalised in a neonatal intensive care unit (NICU) for several weeks or months. Often, parents cannot be permanently present in the ward due to further family or work commitments, the hospital’s restricted visiting hours or because the ward’s spatial capacities may not allow overnight stays. Leaving their infant in the ward is perceived as challenging by parents [1, 2]. During their absence, parents report being anxious regarding their infant’s condition and feel emotionally stressed [2, 3]. As one possible way to support parents in this situation, some NICUs offer a webcam system for parents to watch their infant via livestream when they are not at their infant’s bedside. The webcams of the system that was introduced to the parents in the present study are positioned above the infant’s bed and transmit only the infant’s image, not the immediate surroundings. Additionally, the webcams do not transmit sound data and do not enable communication. To date, this is not standard care in Germany. Qualitative studies indicate that some parents expect webcams to enable them to leave the ward feeling more reassured, as they can check on their infant from home and thus overcome the lack of information during this time [4–7]. Being certain of their infants’ wellbeing, e.g. after visual reassurance via webcam, was reported to decrease mental stress and anxiety in parents [5, 8]. Furthermore, continuous information and transparency regarding their infant’s condition help parents to trust the neonatal healthcare professionals regarding the infant’s care [9, 10].

There is a multitude of definitions of trust with different emphases depending on the discipline for which they have been developed [11]. In this paper, trust is defined based on the interdisciplinary definition by Judith Hupcey et al. ‘as congruence between the expected and the actual behaviour of the trusted person’ ([10]; p. 137) while there is a dependency of the trusting person (i.e., a parent) on the trusted person (i.e., physician or nurse) [11]. When parents are not present in the ward, trust in neonatal healthcare professionals helps them to satisfy their need for security regarding their infant’s condition and care [1, 12]. If parents lack trust in neonatal healthcare professionals, they may attempt to compensate through other means, such as webcam usage. However, some healthcare professionals associate webcam usage with being surveilled by parents [13–15]. Consequently, in daily routines in the ward, healthcare professionals may perceive the parental preference to use a webcam as indicative of insufficient trust, potentially straining the relationship between these parties [5, 14]. These concerns lead to our research question regarding whether the parental preference for webcam usage in NICUs is associated with low trust in physicians and nursing staff.

## Methods

The Neo-CamCare study evaluates the usage of webcams in NICUs and the impact on parents’ psychosocial strains [16]. As part of the study, a retrospective cross-sectional survey was conducted to collect data regarding parental experiences during their infant’s NICU stay and to investigate the parental interest in a webcam system in NICUs.

### Study population and survey procedures

The survey was directed at parents of premature infants with a birth weight below 1500 g. Inclusion criteria were the following ICD-10-GM codes indicating the infant’s birth weight: P07.00 (< 500 g), P07.01 (500 to < 750 g), P07.02 (750 to < 1000 g), P07.10 (1000 to < 1250 g), and P07.11 (1250 to < 1500 g). The study population was further limited to parents of infants aged between six and 18 months at the time of the survey to minimise recall bias while avoiding additional stress for parents shortly after their infant’s birth. Parents whose infant is deceased were not contacted. The participants were selected based on medical accounting data from two German statutory health insurance companies, which dispatched all survey documents. The questionnaires were sent exclusively to mothers, who were asked to pass a second questionnaire to the person who fulfilled the second parental role during the infant’s NICU stay (father or partner). Due to this procedure, it is possible that in some cases both parents of the same infant participated in the survey and in some cases only one parent. However, this cannot be quantified, as data collection was anonymous.

The survey documents comprised a cover letter, one written questionnaire each for the mother and the father/partner with two pre-stamped return envelopes, and an incentive (protective cover for the infant’s vaccination certificate). Furthermore, an information sheet explaining the principles of the webcam system was enclosed.

The participants were asked to return the completed questionnaire anonymously to the evaluating institute (Institute of Medical Sociology, Health Services Research, and Rehabilitation Science [IMVR]). Consequently, the IMVR did not have access to participants’ address data at any time, and therefore, the data collection and analyses are considered anonymous. To increase the response rate, a combined reminder and thank-you letter was sent to all mothers after two weeks. The survey period was from September to December 2020.

### Survey instrument and operationalisation

The questionnaire comprised validated scales as well as items that were developed based on literature review and on interviews conducted during the project [5]. The survey was critically reviewed by a neonatologist, a NICU nurse, and a parent representative. Additionally, the questionnaire was completed on a trial basis and commented on by a mother and a father who satisfied the survey’s inclusion criteria.

The subset of questions regarding webcam preference included enquiries concerning whether a webcam had been offered in the treating ward and whether this offer had been accepted. Parents who had not received a webcam offer were asked whether they would have opted for a webcam if they had received an offer (variable *webcam preference*; options: *yes* = 1 or *no* = 0).

Parental trust was assessed by the Trust in Physicians and Trust in Nursing Staff scales [17]. Both scales consist of five corresponding statements that are rated on a 6-point response format. For our analyses, we generated two variables (*trust in physicians* and *trust in nursing staff*): for each scale a relativised sum score was calculated by adding the rating of the individual statements (*never* = 1, *rarely* = 2, *sometimes* = 3, *often* = 4, *very often* = 5, and *always* = 6) and dividing the sum by 5 for the number of statements.

Given that anxiety reflects a tendency to worry [18], we assumed that trait anxiety might be associated with the desire to permanently monitor the infant to gain a feeling of assurance and control [5]. Therefore, the personality trait anxiety was included in the models as a control variable. It was measured by the German 10-item short version of the State-Trait-Anxiety Inventory by Grimm [19]. The items are rated on an 8-point answering format from *almost never* = 1 to *almost always* = 8 and totalled to determine a sum score (variable *trait anxiety*)*.* To control for general demographic characteristics, we included *parental role* (mother vs. father/partner), *parental age*, and the *educational degree* in the model (please refer to Table 1 for variable specifications).

The (relativised) sum scores for *trust in physicians*, *trust in nursing staff,* and for *trait anxiety* were calculated only for parents who completed all items of the respective scale; all others were excluded from the analyses (number of parents with any incomplete scale item: *n* = 17 [*trust in physicians]*, *n* = 16 [*trust in nursing staff*], and *n* = 32 [*trait anxiety*]).

### Statistical analyses

The question regarding whether a webcam would have been desirable was to be answered only by parents who were not offered a webcam (variable *webcam preference*). Due to the small number of parents who had been offered a webcam (*n* = 25, information missing *n* = 6), we refrained from performing separate analyses for this group regarding their actual decision for or against webcam usage.

*Webcam preference*, *trust in physicians*, *trust in nursing staff*, *trait anxiety*, and demographic information were analysed descriptively. Categorical variables are reported as absolute and relative frequencies in percentage. Continuous variables were tested for normal distribution; as normal distribution was not given, median (Mdn) and interquartile range (IQR) are presented.

We performed binary logistic regression analysis using maximum likelihood estimation to examine the association between trust in neonatal healthcare professionals and the potential preference to use a webcam (dependent variable *webcam preference: yes* or *no*). The overall model fit was assessed based on the likelihood-ratio chi-squared tests. To quantify the extent to which the trust variables improve the model fit compared to a model containing other parental characteristics only (i.e., the control variables mentioned above), two models were calculated: The first model only contained the control variables (*parental role*, *parental age, educational degree,* and *trait anxiety*). In the second model *trust in physicians* and *trust in nursing staff* were added. Continuous variables were centred around the sample mean for the usage within the models. The odds ratios’ p-values were computed using Wald test (z-values). To account for multiple statistical testing, p-values were adjusted using Bonferroni-Holm correction (level of statistical significance < 5%). Variables were checked for multicollinearity by calculating the variance inflation factor (VIF). If a VIF value exceeded 5, the respective variable was to be removed from the model. All analyses were performed via Stata 16.1.

## Results

**Fig. 1:**
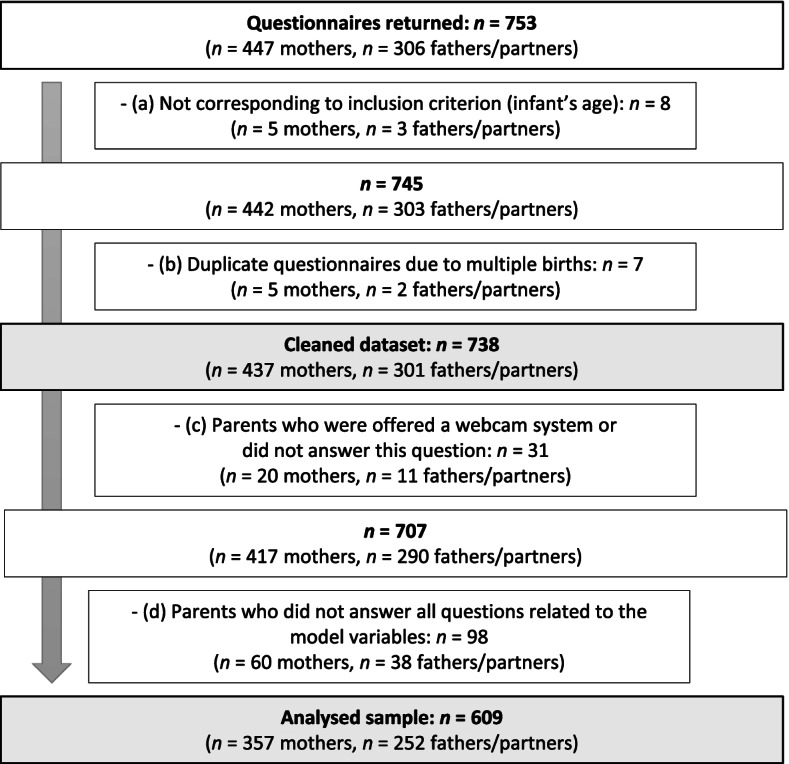
Flowchart of participants from the number of parents who returned questionnaires to the number of parents included in the analyses

Based on medical accounting data from the two statutory health insurance companies, 1001 mothers were eligible for study participation. A total of 753 questionnaires were returned (*n* = 447 mothers, *n* = 306 fathers/partners). The response rate is 44.66% for mothers. For fathers/partners, the response rate could not be calculated because it is unknown how many mothers actually passed the questionnaire to the respective father/partner. For data cleaning (see the flowchart in Fig. 1), we excluded questionnaires from parents whose infant did not meet the inclusion criteria (a) as well as duplicates due to multiple births (b).

In our cleaned dataset, 25 parents (3.39%) were offered the usage of a webcam during their infant’s NICU stay, 707 parents (95.80%) did not receive an offer, and 6 parents (0.81%) did not answer this question. The majority of parents who were offered a webcam system accepted this offer (60.00%, *n* = 15).

To attain our final sample for the regression analysis (analysed sample), we further excluded questionnaires from parents who received a webcam offer or did not answer this question (c) and parents with any incomplete variables that were necessary for the regression models (d). Accordingly, 609 parents (*n* = 357 mothers, *n* = 252 fathers/partners) remained for logistic regression analysis (see Fig. 1).

### Characteristics of the analysed sample

Table 1 presents the characteristics of the parents and their preterm infants for the sample used in the logistic regression analysis. In this sample, parents’ median score for *trust in physicians* was 5.80 (*IQR* = 0.80, *n* = 609). *Trust in nursing staff* was rated slightly lower, with a median score of 5.60 (*IQR* = 1.00, *n* = 609). The median sum score for the control variable *trait anxiety* was 32 (*IQR* = 18, *n* = 609). Internal consistency was satisfactory for all scales in our retrospective sample (*trust in physicians*: α = .904, *trust in nursing staff*: α = .926, *trait anxiety*: α = .859). Regarding the *webcam preference*, 69.13% (*n* = 412) of the parents would have opted for webcam usage if they had received an offer during their infant’s NICU stay. For comparisons of the analysed sample with the cleaned dataset please refer to Table 1 in the Appendix.

**Table 1:** Sample characteristics of parents included in the regression analysis

	*n*	*percent (%)*	*Mdn* (*IQR*); min–max
**Sample description—parents**			
**Parental role**			
Mother	357	58.62	
Father/partner	252	41.38	
**Age (years)**	609		35 (6); 21–58
**Educational degree**			
No completed degree	16	2.63	
Completed vocational or specialist training	302	49.59	
University or college degree	291	47.78	
**Sample description—infants**			
**Birth weight**			
1000 g–1499 g	356	58.46	
< 1000 g	248	40.72	
Missing data	5	0.82	
**Gestational age (weeks)**	606		29 (5); 22–38
**Current age of infant (months)**	604		13 (5); 6–18

### Association of trust and parental preference for a webcam system

The logistic regression model to determine the association of *trust in physicians* and *trust in nursing staff* with the preference to use a webcam system was calculated for parents who did not receive a webcam offer during their infant’s NICU stay. The odds ratios, 95% confidence intervals, p-values, and model statistics of the regression analysis are presented in Table 2.

**Table 2:** Association between trust in physicians and trust in nursing staff and the preference to use a webcam system

	Model 1			Model 2		
	Odds Ratio	95-%-CI	*p*	Odds Ratio	95-%-CI	*p*
**Parental role** ref. mothers						
Fathers/partners	0.628	[0.435,0.906]	.065	0.630	[0.436,0.911]	.098
**Parental age (years)**	0.984	[0.951,1.019]	.690	0.982	[0.948,1.017]	.932
**Educational degree** ref. university or college						
No completed degree	0.347	[0.122,0.989]	.143	0.391	[0.136,1.123]	.393
Completed vocational or specialist training	0.842	[0.589,1.203]	.690	0.851	[0.594,1.221]	.932
**Trait anxiety (sum score)**	1.017	[1.002,1.032]	.102	1.014	[0.998,1.029]	.393
**Trust in physicians (relativised sum score)**				0.654	[0.456,0.937]	.124
**Trust in nursing staff (relativised sum score)**				1.064	[0.783,1.446]	.932
Model statistics	χ^2^(5) = 18.86, *p* = .0020, McFadden’s pseudo R^2^ = .0250, AIC = 745.94, *n* = 609			χ^2^(7) = 25.85, *p* < .001, McFadden’s pseudo R^2^ = .0343, AIC = 742.95, *n* = 609		

The binary logistic regression models are based on all parents who were not offered a webcam system during their infant’s NICU stay (fully completed cases only). The p-value for the model fit was generated using likelihood-ratio chi-squared test; for the odds ratios’ p-values Wald tests (z-values) were used. *CI* Confidence interval for odds ratios, *p* p-value for odds ratios after the Bonferroni-Holm correction, *ref.* Reference category, *pseudo R*^*2*^ McFadden’s-R^2^, *AIC* Akaike information criterion

In our analysis, no significant association was found for *trust in physicians* and the preference to use a webcam system or for *trust in nursing staff* and webcam preference. Furthermore, a comparison of the two models reveals that the trust variables only ameliorated the model fit to a small extent with an overall small pseudo *R*^*2*^ (pseudo *R*^*2*^_Model1_ = .0250 vs. pseudo *R*^*2*^_Model2_ = .0343). None of the remaining parental factors were significantly associated with the webcam preference.

## Discussion

As interest increases in implementing webcams in NICUs, it is important to know which aspects motivate parents to opt for webcam usage. The present study focused on the association between trust in physicians and nursing staff and webcam preference.

Our results indicate that parental trust in physicians and nursing staff is generally very high and that trust does not appear to be associated with parental preference to watch their infant via webcam. Furthermore, parental role, age, education, and trait anxiety were not associated with the webcam preference either. Hence, other underlying factors must exist, such as parents’ beliefs regarding the advantages or disadvantages of webcam usage. Possible advantages mentioned by parents include improved involvement in their infant’s care and the need to check their infant’s condition when not in the ward [5].

The missing association between trust and webcam preference aligns with previous research, which suggests that trust and vigilant behaviour can exist simultaneously. For adult patients and their families mistrust in healthcare professionals may result in continuous vigilance and critical observation of care [20, 21]; however, parents of young children do not feel that trust and vigilant behaviour are contradictory [10]. Virginia Thompson et al. argue in their model regarding trust development that vigilant behaviour and the need for continuous information are inherent in the parental role and relate to fulfilling the parents’ needs rather than symbolising mistrust [10]. Therefore, parents’ preference to use a webcam may indicate an anticipated increase in information concerning their infant’s condition [5]. Notably, the effect of webcam usage on parents may concern various aspects of parental wellbeing and could be both positive and negative. Jennifer Weber et al. do not find a significant difference between parents with and without webcam usage in one NICU regarding the perceived information status or feelings of anxiety when separated from their infant [22]. Other researchers report that parents feel reassured and less stressed concerning the separation when they use a webcam [6, 23, 24]. However, it can be expected that webcams provide this reassuring effect only if the infant appears to be well through the webcam. A preterm infant’s mere physical appearance can be a stressor [25–27] and witnessing their infant in discomfort or in pain and being unable to intervene from afar may result in additional parental stress and feelings of powerlessness [5, 7].

Such unsettling observations through a webcam may impair the trusting bond between parents and neonatal healthcare professionals, if parents acquire the subjective impression that their infant is not being cared for sufficiently [5]. Therefore, to avoid straining the relationship between parents and neonatal healthcare professionals, the risk of witnessing distressing situations must be openly addressed when informing parents about webcam usage. Regardless of any webcam provision, neonatal healthcare professionals should place a high value on parental information in their daily work. When webcams are available, up-to-date and transparent information on their infant’s condition and treatment remain crucial, as they allow parents to judge what they see through the webcam.

We intended to capture a clear statement on webcam preference by using a dichotomous answering format. Therefore, information about possible gradations within the webcam preference is not available. Trust was measured retrospectively in this study; consequently, recall bias cannot be completely eliminated in our sample. Further, although health insurance is compulsory in Germany and the two participating health insurance companies are among the largest in Germany, it cannot be entirely ruled out that our sample is biased due to the membership in one of these specific health insurance companies. Moreover, we did not collect information on the infants’ medical outcomes or parents’ character traits other than anxiety. However, severe complications or parental coping behaviour may also be influential in the evaluation of trust and webcam preference. Even though only 44.66% of mothers responded to our survey, this does not necessarily entail a lower external validity. As Susan Morton et al. elaborate, response rate is not a sufficient criterion for assessing representativeness [28]. A comparison of the study participants with parents who had been invited but declined participation would have been favourable but was not possible in our study due to the fact that data collection was anonymous. It is to be noted that parents of a deceased infant were not contacted by the health insurance companies, so their perspective is not included in our results. Nevertheless, it is important to point out the strengths our sample. By recruiting the participants through the health insurance companies, a selection bias due to place of residence or hospital could be avoided and we obtained a large, nationwide sample. Due to these strengths, we consider our results to be valuable, despite the limitations listed.

Furthermore, we did not intend to determine causality in the relationship between parental trust and webcam preference. The development of trust in nursing staff is related to receiving continuous information and perpetually comparing the expected and actual behaviour of healthcare professionals [10, 11]. Both are supported by webcam usage. Previous research indicates that webcam usage can help parents *to build* trust in nursing staff [9]. In this case, webcam usage could influence the development and maintenance of a trusting bond between parents and neonatal healthcare professionals, as outlined above. Therefore, webcam usage in NICUs and the possible causal influence on trust will be further investigated in the framework of the project Neo-CamCare using a prospective study design.

## Conclusion

In our analysed sample, more than three-quarters of parents would have chosen to use a webcam if one had been offered. Trust in physicians and nursing staff is not significantly associated with this preference. Consequently, the parental preference to use a webcam should not be considered an indicator of lacking trust in neonatal healthcare professionals.

## Supplementary Information

**Additional file 1: Appendix Table 1.**

## Data Availability

The datasets used or analysed during the current study are available from the corresponding author upon reasonable request.

## Abbreviations

ICD-10-GM: International Statistical Classification of Diseases and Related Health Problems, 10th revision, German Modification
IMVR: Institute of Medical Sociology, Health Services Research, and Rehabilitation Science
IQR: Interquartile range
Mdn: Median
NICU: Neonatal intensive care unit
VIF: Variance Inflation Factor
