# Supplementary material for: Parental preference for webcams in neonatal intensive care units: an indicator of lacking trust?
Source: BMC Pediatr. 2022 Jul 11;22:406. doi: 10.1186/s12887-022-03456-2 (PMC9277807; doi:10.1186/s12887-022-03456-2)
Supplement: Supplementary file 1 — Additional file 1: Appendix Table 1. [file 12887_2022_3456_MOESM1_ESM.docx]

**Appendix to ‘Parental preference for webcams in neonatal intensive care units: An indicator of lacking trust?’**

Laura Mause,**^1^** Alinda Reimer,**^1^** Jan Hoffmann,**^1^** Till Dresbach,^2^ Dirk Horenkamp-Sonntag,^3^ Melanie Klein,^4^ Nadine Scholten**^1^** on behalf of Neo-CamCare

**^1^ Institute of Medical Sociology, Health Services Research, and Rehabilitation Science, Faculty of Human Sciences and Faculty of Medicine, University of Cologne and University Hospital Cologne, Cologne, Germany**

^2^ Department of Neonatology and Pediatric Intensive Care, Children’s Hospital, University Hospital Bonn, Bonn, Germany

^3^ Techniker Krankenkasse, Healthcare Management, Hamburg, Germany

^4^ DAK Gesundheit, Hamburg, Germany

Table 3. Comparison of the cleaned dataset and the analysed sample.

|  | **Cleaned dataset (*n* = 738)** | | |  | **Analysed sample (*n* = 609)** | | |
| --- | --- | --- | --- | --- | --- | --- | --- |
|  | *n* | *percent (%)* | *M ± SD*  *Mdn* (min–max) |  | *n* | *percent (%)* | *M ± SD*  *Mdn* (min–max) |
| **Sample characteristics—parents** |  |  |  |  |  |  |  |
| **Parental role** |  |  |  |  |  |  |  |
| Mother | 437 | 59.21 |  |  | 357 | 58.62 |  |
| Father/partner | 301 | 40.79 |  |  | 252 | 41.38 |  |
| **Age (years)** | 736 |  | 35.25 ± 5.11 |  | 609 |  | 35.30 ± 5.14 |
|  |  |  | 35 (2–58) |  |  |  | 35 (21–58) |
| **Educational degree** |  |  |  |  |  |  |  |
| No completed degree | 25 | 3.39 |  |  | 16 | 2.63 |  |
| Completed vocational or specialist training | 360 | 48.78 |  |  | 302 | 49.59 |  |
| University or college degree | 346 | 46.88 |  |  | 291 | 47.78 |  |
| Missing data | 7 | 0.95 |  |  | - | - |  |
| **Trust in physicians (relativised sum score)** | 718 |  | 5.51 ± 0.67 |  | 609 |  | 5.50 ± 0.67 |
|  |  |  | 5.8 (1.80–6.00) |  |  |  | 5.80 (2.00–6.00) |
| **Trust in nursing staff (relativised sum score)** | 718 |  | 5.34 ± 0.72 |  | 609 |  | 5.34 ± 0.73 |
|  |  |  | 5.60 (2.40–6.00) |  |  |  | 5.60 (2.40–6.00) |
| **Trait anxiety (sum score)** | 702 |  | 32.84 ± 12.12 |  | 609 |  | 33.13 ± 12.29 |
|  |  |  | 32 (10.00–73.00) |  |  |  | 32 (10.00–73.00) |
| **Sample characteristics—infants** |  |  |  |  |  |  |  |
| **Birth weight** |  |  |  |  |  |  |  |
| 1,000 g–1,499 g | 426 | 57.72 |  |  | 356 | 58.46 |  |
| < 1,000 g | 304 | 41.19 |  |  | 248 | 40.72 |  |
| Missing data | 8 | 1.08 |  |  | 5 | 0.82 |  |
| **Gestational age (weeks)** | 732 |  | 29.23 ± 2.88 |  | 606 |  | 29.27 ± 2.92 |
|  |  |  | 29 (22–38) |  |  |  | 29 (22–38) |
| **Current age of infant (months)** | 731 |  | 12.85 ± 3.12 |  | 604 |  | 12.95 ± 3.07 |
|  |  |  | 13 (6–18) |  |  |  | 13 (6–18) |

Compare Figure 1 in the main text for the flowchart concerning the two samples.
